# Chromosome-Level Assembly of the Atlantic Silverside Genome Reveals Extreme Levels of Sequence Diversity and Structural Genetic Variation

**DOI:** 10.1093/gbe/evab098

**Published:** 2021-05-08

**Authors:** Anna Tigano, Arne Jacobs, Aryn P Wilder, Ankita Nand, Ye Zhan, Job Dekker, Nina Overgaard Therkildsen

**Affiliations:** 1 Department of Natural Resources, Cornell University, Ithaca, New York, USA; 2 Department of Molecular, Cellular and Biomedical Sciences, University of New Hampshire, Durham, New Hampshire, USA; 3 Conservation Genetics, San Diego Zoo Global, Escondido, California, USA; 4 Program in Systems Biology, University of Massachusetts Medical School, Worcester, Massachusetts, USA; 5 Howard Hughes Medical Institute, Chevy Chase, Maryland, USA

**Keywords:** fish, genome assembly, heterozygosity, Hi-C, inversions, nucleotide diversity

## Abstract

The levels and distribution of standing genetic variation in a genome can provide a wealth of insights about the adaptive potential, demographic history, and genome structure of a population or species. As structural variants are increasingly associated with traits important for adaptation and speciation, investigating both sequence and structural variation is essential for wholly tapping this potential. Using a combination of shotgun sequencing, 10x Genomics linked reads and proximity-ligation data (Chicago and Hi-C), we produced and annotated a chromosome-level genome assembly for the Atlantic silverside (*Menidia menidia*)—an established ecological model for studying the phenotypic effects of natural and artificial selection—and examined patterns of genomic variation across two individuals sampled from different populations with divergent local adaptations. Levels of diversity varied substantially across each chromosome, consistently being highly elevated near the ends (presumably near telomeric regions) and dipping to near zero around putative centromeres. Overall, our estimate of the genome-wide average heterozygosity in the Atlantic silverside is among the highest reported for a fish, or any vertebrate (1.32–1.76% depending on inference method and sample). Furthermore, we also found extreme levels of structural variation, affecting ∼23% of the total genome sequence, including multiple large inversions (> 1 Mb and up to 12.6 Mb) associated with previously identified haploblocks showing strong differentiation between locally adapted populations. These extreme levels of standing genetic variation are likely associated with large effective population sizes and may help explain the remarkable adaptive divergence among populations of the Atlantic silverside.

Significance

Standing genetic variation can provide insights about the evolutionary history of a species. The chromosome-level genome assembly for the Atlantic silverside, an ecological model for the study of adaptation, allows us to analyze sequence and structural variation jointly, and thus to start understanding how adaptation and demography shape genome-wide patterns of variation. Our analyses reveal extreme levels of standing genetic variation, with a sequence variant every 57–75 bases and over 20% of the genome affected by structural variants, and that large blocks of differentiation previously associated with local adaptations coincide with large chromosomal inversions. These results are consistent with very large population sizes and remarkable variation in local adaptations across the Atlantic silverside geographic range.

## Introduction

Standing genetic variation is widely recognized as the main source of adaptation (Barrett and Schluter 2008; Tigano and Friesen 2016) and is important for natural populations to maximize their potential to adapt to changes in their environment. As genetic diversity results from the interplay of mutation, selection, drift, and gene flow, the levels and patterns of standing genetic variation found within a species can provide important insights not only about its adaptive potential but also about its demographic and evolutionary history.

Standing genetic variation encompasses both sequence and structural variation, including changes in DNA sequence, and in the position, orientation, and number of copies of sequence, though the latter has often been neglected until recently. Structural variation has, however, been associated both directly and indirectly with many traits involved in speciation and adaptation and is abundant in the few genomes in which it has been catalogued (Wellenreuther and Bernatchez 2018; Catanach et al. 2019; Lucek et al. 2019; Mérot et al. 2020; Tigano et al. 2020; Weissensteiner et al. 2020). Structural variants (SVs) can directly affect phenotypic traits (e.g., Van’t Hof et al. 2016), or may promote the maintenance of divergent haplotypes between locally adapted populations or groups (e.g., ecotypes or morphs) within single populations via recombination suppression (e.g., Faria et al. 2019; Kess et al. 2020). Structural variation is therefore a key component of standing genetic variation. To better quantify levels of standing variation and understand how demographic and evolutionary factors contribute to their distribution across the genome, we need to examine sequence and structural variation jointly. A high-quality reference genome for the species of interest is therefore fundamental as we need both broad coverage and high contiguity to accurately assess both sequence and structural variation.

The Atlantic silverside (*Menidia menidia*), a small coastal fish distributed along the Atlantic coast of North America, shows a remarkable degree of local adaptation in a suite of traits, including growth rate, number of vertebrae, and temperature-dependent sex determination (Hice et al. 2012), that are associated with strong environmental gradients across its wide latitudinal range. This species also provided the first discovery of temperature-dependent sex determination in fishes (Conover and Kynard 1981) and was one of the first species in which countergradient phenotypic variation (i.e., when phenotypic variation on a trait is minimized by the effect of the environment counteracting the genetic predisposition across environmental clines) was documented (Conover and Present 1990). Through extensive prior work, the Atlantic silverside has, in fact, become an important ecological model to study the phenotypic effects of selection, both natural and artificial, in the wild and under controlled conditions in the lab (Conover and Munch 2002; Conover et al. 2005; Hice et al. 2012). In one iconic experiment, wild-caught Atlantic silversides were subjected to different size-selective regimes to investigate the potential of fisheries to induce evolutionary change in harvested species (Conover and Munch 2002). Seventeen years later, exome analysis of fish from that experiment identified substantial allele frequency shifts associated with rapid phenotypic shifts in growth rates (Therkildsen et al. 2019). In the absence of a reference genome, genomic reads were mapped to the silverside reference transcriptome, so only protein-coding regions of the genome were analyzed (“in silico” exome capture). Yet, anchoring the transcriptome contigs to the medaka (*Oryzias latipes*) chromosome-level reference genome revealed that the most conspicuous allele frequency shifts clustered into a single block on chromosome 24, where more than 9,000 single nucleotide polymorphisms (SNPs) in strong linkage disequilibrium (LD) increased from low (<0.05) to high frequency (∼0.6) in only five generations. Additional data from natural populations across the geographical distribution of the species showed that this same block, likely spanning several Mb of the chromosome, was fixed for opposite haplotypes among wild silverside populations that naturally differ in growth rates (Conover and Present 1990; Conover and Munch 2002; Therkildsen et al. 2019). Moreover, three additional blocks comprising hundreds of genes in high LD were found to be segregating among the natural populations—with each LD block (“haploblocks” hereafter) mapping predominantly to unique medaka chromosomes—and were enriched for genes with functions associated with known local adaptations (Wilder et al. 2020). Similar to the haploblock on chromosome 24, opposite haplotypes in these haploblocks were nearly fixed between natural populations that otherwise showed low genome-wide pairwise differentiation. In populations where both northern and southern haplotypes within these blocks occur, heterozygous individuals were found in Hardy–Weinberg proportions, suggesting that they do not confer obvious hybrid incompatibilities, at least in F1 crosses (Therkildsen et al. 2019; Wilder et al. 2020). Furthermore, strong LD between genes in these blocks suggests that local recombination suppression, possibly due to inversions, and natural selection have maintained these divergent haploblocks in the face of gene flow. It thus appears that large haploblocks play an important role in maintaining local adaptations in the Atlantic silverside, although the exact extent of the genome spanned by these haploblocks and the genomic mechanisms maintaining LD are unknown.

Given the wealth of ecological information available for the Atlantic silverside and its potential as an evolutionary model to study adaptation and fishery-induced evolutionary change, developing genomic resources for this species is timely and holds great potential for addressing many pressing questions in evolutionary and conservation biology. Previous population genomic analyses based on the transcriptome reference anchored to the medaka genome were limited to the coding genes and, given the unknown degree of synteny between the Atlantic silverside and the medaka, which are 91 million year divergent (timetree.org), it was uncertain how variants relevant to adaptation and fishery-induced selection clustered in the genome. To enable analysis of both coding and noncoding regions, to accurately estimate the levels and genomic distribution of standing genetic variation, both sequence and structural, and to reconstruct the specific genomic structure of the Atlantic silverside genome, we produced a chromosome-level genome assembly for the species using a combination of genomic approaches. Because of known geographic differentiation, we estimated levels of sequence variation within genomes from both the southern and northern parts of the distribution range and characterized standing structural variation between these two genomes. Finally, we tested whether the haploblocks identified on four different chromosomes between southern and northern populations were associated with large inversions as the patterns of differentiation and LD suggested (Therkildsen et al. 2019; Wilder et al. 2020). Our work illustrates the wealth of information that can be obtained from the analysis of one or two genomes in the presence of a high-quality reference sequence, and shows 1) that the Atlantic silverside has one of the highest levels of nucleotide diversity among vertebrates, and the highest levels of structural variation reported so far within a species, and 2) that both the chromosome structure, including centromeres and telomeres, and SVs appear to affect the distribution of diversity across the genome in this species. These results taken together highlight the importance of high-quality genomic resources as they enable the joint analysis of sequence and structural variation at the whole-genome level.

## Results

### Genome Assembly and Assessment of Completeness

We built a reference genome for the Atlantic silverside using a combination of 10x Genomics linked-reads technology (10x Genomics, Pleasanton, CA) and proximity-ligation data generated with Chicago^®^ (Putnam et al. 2016) and Dovetail™ Hi-C (Lieberman-Aiden et al. 2009) protocols. With the 10x data, we obtained the best draft assembly (based on a combination of summary statistics, see supplementary table S1, Supplementary Material online) when we downsampled to 270 million reads as input to *Supernova* (Weisenfeld et al. 2017). Assembly contiguity increased more than 2-fold after incorporating Dovetail Chicago data (scaffold N50 from 1.3 to 2.9 Mb) and more than 10-fold with Dovetail Hi-C data (scaffold N50 = 18.2 Mb). Summary statistics for each of the intermediate genome assemblies (10x, Dovetail Chicago, and Dovetail Hi-C) are presented in table 1. The final assembly—including scaffolds longer than 1 kb only—was 620 Mb in total length. Overall, this assembly showed high contiguity, high completeness and a low proportion of gaps (table 1). Analysis of the presence of BUSCO genes showed that only 5.9% of the Actinopterygii gene set was missing from the assembly. Although the number of missing genes did not decrease dramatically from the 10x assembly to the final assembly (from 6.6% to 5.9%), the addition of proximity-ligation data (Chicago and Hi-C) increased the number of complete genes (from 88.1% to 89.6%) and decreased the number of duplicated (from 4.1% to 2.9%) and fragmented genes (from 5.3% to 4.5%). Contiguity did not come at the cost of increased gappiness, as stretches of N’s made up only 3% of the final assembly. The reduction of the final assembly to its longest 27 scaffolds > 1 Mb, which we call the “chromosome assembly” based on chromosomal synteny between the Atlantic silverside and medaka and Hi-C data (see Repeat and Gene Annotation), resulted in a 25% reduction in sequence but increased missing genes by only 3.1% and reduced duplicated genes to 1.9%. K-mer analyses based on raw data from the reference genome estimated a genome size of 554 Mb, 76 Mb shorter than the final assembly and 88 Mb longer than the chromosome assembly. Our “chromosome assembly” is therefore unlikely to be 100% complete, but the modest loss of gene content in the final reduction step suggests that the unassembled regions are likely to represent gene-poor repetitive sequence.

**Table 1:** Summary statistics for each of the intermediate and final assemblies of the reference genome from Georgia

	10x	Dovetail Chicago	Dovetail Hi-C	Final Assembly	Chromosome Assembly
Total length	645.45 Mb	647.32 Mb	647.39 Mb	620.04 Mb	465.69 Mb
Longest scaffold	12,248,921 bp	12,871,938 bp	26,678,928 bp	26,678,928 bp	26,678,928 bp
Number of scaffolds	99,541	80,990	80,312	42,220	27
Number of scaffolds > 1 kb	61,451	42,898	42,220	42,220	27
Contig N50	39.55 kb	39.51 kb	39.51 kb	105.76 kb	202.88 kb
Scaffold L50/N50	83/1.328 Mb	42/2.936 Mb	16/18.159 Mb	15/18.199 Mb	11/19.68 Mb
% gaps	2.69%	2.97%	2.98%	3.08%	3.00%
BUSCOs^a^ (*n* = 4,584)	C: 88.1%; F: 5.3%; M: 6.6%	C: 89.5%; F: 4.6%; M: 5.9%	C: 89.6%; F: 4.8%; M: 5.6%	C: 89.6%; F: 4.5%; M: 5.9%	C: 88.3%, F: 2.7%; M: 9.0%

Note.—“10x” refers to the draft assembly based only on 10x linked reads including scaffolds > 500 bp, “Dovetail Chicago” refers to the 10x assembly improved with Dovetail Chicago library data, and “Dovetail Hi-C” refers to the 10x assembly improved with both Dovetail Chicago and Hi-C data. The “Final assembly” represents the Dovetail Hi-C assembly but including only scaffolds > 1 kb, and the “Chromosome assembly” is the subset of scaffolds > 1 Mb from the “Final assembly.”

a C, complete; F, fragmented; M, missing.

### Repeat and Gene Annotation

Repeat annotation based on a combination of a de novo developed species-specific libraries and a database of known repeats in teleosts suggested that repetitive elements made up 17.73% of the Atlantic silverside genome (“final assembly”), in line with expectations based on fish species with similar genome sizes (Yuan et al. 2018). The biggest proportion of these repeats was made up of interspersed repeats (15.34% of the genome), while transposable elements constituted 8.83% of the genome overall (0.90% of SINEs, 2.79% of LINEs, 1.54% of LTR elements, and 3.60% of DNA elements). Our gene prediction pipeline identified a total of 21,644 protein-coding genes, a number consistent with annotated gene counts in other fish species (Lehmann et al. 2019; Ozerov et al. 2018). Analysis in *Blast2GO* (Götz et al. 2008) based on homology and *InterProScan2* (Zdobnov and Apweiler 2001) resulted in functional annotation of 17,602 out of the 21,644 protein-coding genes (81.3%; https://github.com/atigano/Menidia_menidia_genome/annotation/). Further, *InterProScan2* detected annotations (*Panther* or *PFAM*) for an additional 1,511 genes, for which no BLAST results were obtained.

### Synteny with Medaka

**Fig. 1.**
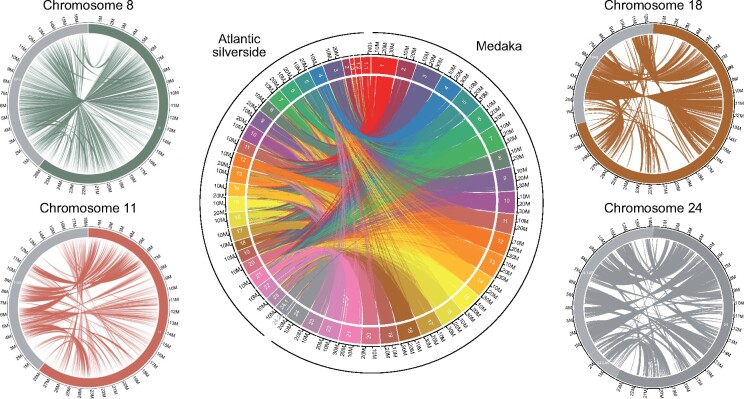
Circos plots showing synteny between the Atlantic silverside and medaka across all chromosomes (center) and in the four chromosomes (left and right) with large haploblocks on the sides. Chromosomes are color-coded consistently among plots and the colored portion (dark gray for chromosome 24) of the smaller plots refer to the medaka sequences on the right, whereas the light gray portion to the Atlantic silverside sequences on the left. Alignments shorter than 500 bp were excluded. Supplementary figure S1, Supplementary Material online shows plots for the remaining chromosomes. Note that the consistently shorter length of the Atlantic silverside genome is consistent with a lower overall estimate of genome size (554 Mb based on k-mer analysis compared with the 700 Mb of the assembled medaka genome). The three and two scaffolds making up chromosomes 1 and 24, respectively, are represented separately here and denoted by decimal suffixes (e.g., 1.1 and 24.1).

The chromosome-level genome assembly of medaka (*O.latipes*) was used by Therkildsen et al. (2019) and Wilder et al. (2020) to order and orient contigs of the Atlantic silverside transcriptome (Therkildsen and Baumann 2020) but the degree of synteny between the two species was unknown. Alignment of the 27 largest Atlantic silverside scaffolds to the medaka genome revealed a high degree of synteny conservation, especially considering the evolutionary distance between the two species. Each Atlantic silverside scaffold mapped mostly to only a single medaka chromosome, and 22 of the 24 medaka chromosomes had matches with only one Atlantic silverside scaffold each (fig. 1). Two medaka chromosomes, 1 and 24, had matches with three and two silverside scaffolds, respectively (fig. 1). Based on these results, karyotype data confirming that the medaka and silverside have the same number of chromosomes (Uwa and Ojima 1981; Warkentine et al. 1987), and additional support from Hi-C data from a different individual from Connecticut (details below), we ordered and renamed the Atlantic silverside scaffolds according to the orthologous medaka chromosomes. We grouped the three and two scaffolds that mapped to medaka chromosomes 1 and 24, respectively, into one pseudo-chromosome each and renamed them accordingly. Although we did not observe large interchromosomal rearrangements in the alignment of the silverside and medaka genomes (fig. 1), intrachromosomal rearrangements were common (fig. 1 and supplementary fig. S1, Supplementary Material online). The most conspicuous chromosomal rearrangements were large inversions, intrachromosomal translocations and duplications (fig. 1 and supplementary fig. S1, Supplementary Material online). On chromosomes 8, 11, 18, and 24, where large geographically differentiated haploblocks were identified among natural silverside populations (Wilder et al. 2020), several translocations and inversions were evident, indicating poor intrachromosomal synteny (fig. 1). This was also the case for most of the other chromosomes (supplementary fig. S1, Supplementary Material online).

### Sequence and Structural Standing Variation

**Fig. 2.**
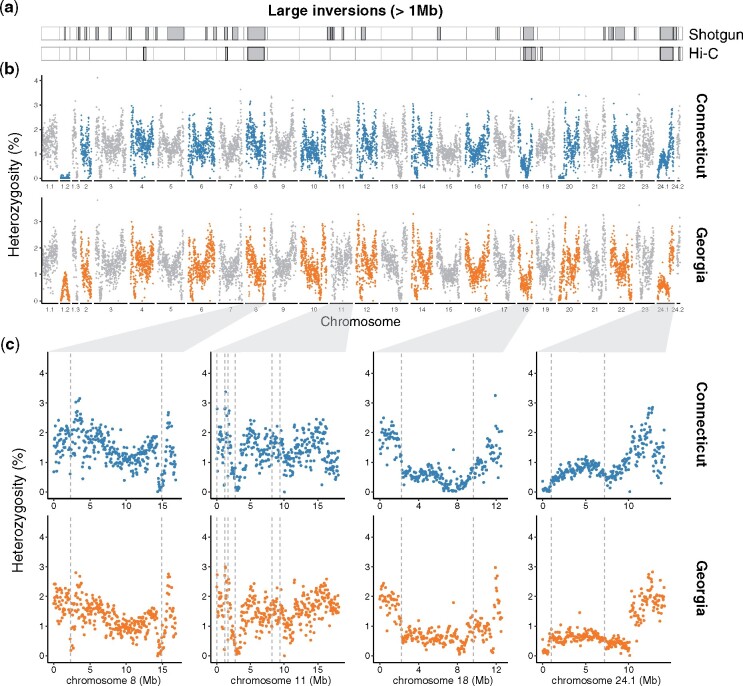
The genomic landscape of structural and sequence variation in Connecticut and Georgia. (*a*) Large inversions (> 1 Mb) as identified from shotgun and Hi-C data from two different individuals from Connecticut mapped to the reference genome from Georgia. (*b*) Manhattan plots showing the genomic landscape of variation in heterozygosity in 50 kb moving windows across single genomes from Connecticut and Georgia where the alternating colors are used to distinguish adjacent chromosomes. The three and two scaffolds making up chromosomes 1 and 24, respectively, are represented separately here and denoted by decimal suffixes. (*c*) Enlarged Manhattan plots for each of the four chromosomes with large haploblocks and inversions. Dashed vertical line represents the breakpoints of the large inversions as identified by *Delly2* with the shotgun data.

The reference genome was sequenced from two lab-reared offspring of wild parents caught in the southern end of the species distribution range (Georgia, USA). To compare patterns of diversity across different populations known to exhibit divergent local adaptations and estimate sequence divergence between the two populations, we also generated a separate draft assembly from an individual sampled from a more northern population (Connecticut, USA) and sequenced with a combination of standard short-insert Illumina whole-genome sequencing to ∼74× coverage and mate-pair sequencing (see supplementary table S2, Supplementary Material online for details). The draft genome (scaffolds >1 kb) from Connecticut had an N50 of 1.67 Mb with an assembly size of 481 Mb, 22% shorter than the 10x draft assembly from Georgia (see assembly stats in supplementary table S3, Supplementary Material online). K-mer analyses based on raw short-read data from one individual from each population resulted in similar estimates of genome sizes and levels of heterozygosity: genome size estimates differed by 20 Mb (554 and 535 Mb in the Georgia and Connecticut individuals, respectively) and heterozygosity estimates differed by 0.09% (1.76% and 1.67% in Georgia and Connecticut, respectively; table 2). Direct estimates of heterozygosity, i.e., based on the number of called heterozygous sites in the genome, were slightly lower and differed by 0.14% between individuals (1.32% and 1.46% in Georgia and Connecticut, respectively; table 2). Together, these estimates concordantly indicate that standing sequence variation in this species is very high (Kajitani et al. 2014), with 1 in every ∼66 bp being heterozygous within each individual, comparable to the European sardine and two eel species, but otherwise higher than most fish species for which estimates are available (table 2). Heterozygosity varied substantially across the genome. Within each chromosome, heterozygosity was highest toward the edges (presumably in areas corresponding to telomeres), decreased towards the center in a U-shape fashion, and showed a deep dip in which the number of heterozygous sites approached zero, a pattern consistent with putative locations of centromeres (fig. 2*b*). Additionally, the proportions of variable sites in coding regions were ∼50% of whole-genome level estimates (0.68% and 0.70% in Georgia and Connecticut, respectively). Swaths of low heterozygosity were particularly evident on chromosomes 18 and 24, two of the four chromosomes with highly differentiated haploblocks (Fig. 2*a* and *b*).

**Table 2:** Examples of heterozygosity levels in single fish genomes, estimated either with GenomeScope from raw sequencing data or through direct calling of heterozygous sites

Common Name	Scientific Name	Heterozygosity (%)	Method	Reference
**Atlantic silverside (GA)**	***Menidia menidia***	**1.76**	GenomeScope	**This study**
**Atlantic silverside (CT)**	***Menidia menidia***	**1.67**	GenomeScope	**This study**
European sardine	*Sardina pilchardus*	1.60–1.75	GenomeScope	Machado et al. (2018)
American eel	*Anguilla rostrata*	1.5–1.6	GenomeScope	Jansen et al. (2017)
European eel	*Anguilla anguilla*	1.48–1.59	GenomeScope	Jansen et al. (2017)
**Atlantic silverside (CT)**	***Menidia menidia***	**1.46**	**Variant calling**	**This study**
Pearlscale pygmy angelfish	*Centropyge vrolikii*	1.36	GenomeScope	Fernandez-Silva et al. (2018)
**Atlantic silverside (GA)**	***Menidia menidia***	**1.32**	**Variant calling**	**This study**
Marine medaka	*Oryzias melastigma*	1.19	GenomeScope	Kim et al. (2018)
Large yellow croaker	*Larimichthys crocea*	1.06	GenomeScope	Mu et al. (2018)
Javafish medaka	*Oryzias javanicus*	0.96	GenomeScope	Takehana et al. (2020)
Greater amberjack	*Seriola dumerili*	0.65	GenomeScope	Sarropoulou et al. (2017)
Clownfish	*Amphiprion ocellaris*	0.60	GenomeScope	Tan et al. (2018)
Hilsa shad	*Tenualosa ilisha*	0.58–0.66	GenomeScope	Mollah et al. (2019)
Whitefish	*Coregonus sp. “Balchen”*	0.44	GenomeScope	De-Kayne et al. (2020)
Corkwing wrasse	*Symphodus melops*	0.40	GenomeScope	Mattingsdal et al. (2018)
Herring	*Clupea harengus*	0.32	Variant calling	Martinez Barrio et al. (2016)
Golden pompano	*Trachinotus ovatus*	0.31	GenomeScope	Zhang et al. (2019)
Coelacanth	*Latimeria chalumnae*	0.28	Variant calling	Amemiya et al. (2013)
NA	*Lucifuga gibarensis*	0.26	GenomeScope	Policarpo et al. (2020)
Eurasian perch	*Perca fluviatilis*	0.24–0.28	GenomeScope	Ozerov et al. (2018)
Atlantic cod	*Gadus morhua*	0.20	Variant calling	Star et al. (2011)
Big-eye mandarin Fish	*Siniperca knerii*	0.16	GenomeScope	Lu et al. (2020)
Threespine stickleback	*Gasteosteus aculeatus*	0.14	Variant calling	Jones et al. (2012)
Pikeperch	*Sander lucioperca*	0.14	GenomeScope	Nguinkal et al. (2019)
African arowana	*Heterotis niloticus*	0.13	GenomeScope	Hao et al. (2020)
Orange clownfish	*Amphiprion percula*	0.12	GenomeScope	Lehmann et al. (2019)
Murray cod	*Maccullochella peelii*	0.10	GenomeScope	Austin et al. (2017)
Toothed Cuban cusk-eel	*Lucifuga dentata*	0.10	GenomeScope	Policarpo et al. (2020)

Note.— The reported estimates of heterozygosity are expressed in percentages, i.e., the number of heterozygous sites per 100 bp, and can be converted to mutations/bp, in which π estimates are generally expressed, by dividing by 100. In bold are the estimates for the Atlantic silverside from this study. 'GA' stands for Georgia and 'CT' stands for Connecticut, the two locations of origin of the individuals analyzed.

We identified a total of 4,900 SVs—including insertions, deletions, duplications, and inversions (table 3 and supplementary file, Supplementary Material online)—between the reference genome generated from Georgia samples and the shotgun-sequenced individual from Connecticut with *Delly2* (Rausch et al. 2012). The identified insertions were small (42–83 bp) and affected a negligible proportion of the genome, while variants classified as deletions were larger and more abundant, covering 15% of the genome sequence (table 3). As an insertion in one genome corresponds to a deletion in the other genome depending on which individual is used as reference, the discrepancy between insertions and deletions is an artifact of mapping short-read sequences to a single reference, i.e., inserted sequences found only in Connecticut are not present in the reference and thus are not mapped. These results highlight the difficulties in identifying insertions and estimating their sizes from short reads. Our analysis detected a small number of duplications, covering only 0.1% of the genome (table 3). Note, however, that we excluded SV calls that were supported by more than 100 reads to exclude repetitive elements from the analysis. Therefore, duplications may be more abundant than currently estimated. In contrast, we identified 662 inversions ranging from 203 bp to 12.6 Mb in size. In total, inversions affected 109 Mb, or 23%, of the reference chromosome assembly (table 3). Twenty-nine inversions were larger than 1 Mb, and five larger than 5 Mb (genomic locations in fig. 2*a* and in supplementary file, Supplementary Material online). *Delly2* identified large inversions (> 1 Mb) on all four chromosomes with previously identified haploblocks: The largest inversion (∼12 Mb) was identified on chromosome 8; chromosome 11 had two 1.2-Mb inversions that were 7 Mb apart; chromosome 18 had a 7.4 Mb inversion and chromosome 24 had two inversions, the first one spanning 9.4 Mb and followed by another one at a distance of 76 kb, spanning 2.3 Mb (fig. 2*a*).

**Table 3:** Summary of intraspecific SVs identified in the Atlantic silverside by mapping sequence data from an individual from Connecticut to the reference genome from Georgia, and their features

SV Type	Number of Variants	Size Range (bp)	Sequence Affected (kb)	% Genome Affected
Insertions	299	42–83	18	<0.01
Deletions	3,905	38–9,740,501	71,754	15
Duplications	34	110–150,263	479	0.1
Inversions	662	203–12,585,625	109,201	23

**Fig. 3.**
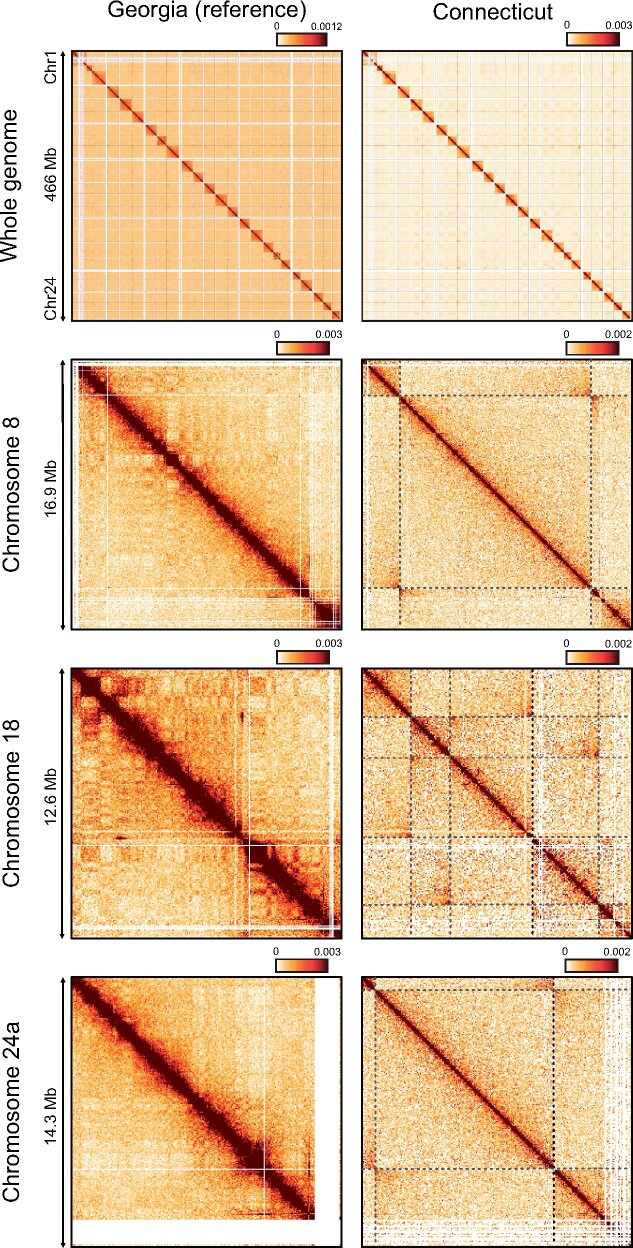
Hi-C contact maps of data mapped to the chromosome assembly from Georgia. Maps on the left show Hi-C data obtained from the same Georgia individual used to generate the reference assembly (mapped to self), maps on the right show data obtained from a Connecticut individual. Maps in the top panel show data for all the chromosomes binned in 100 kb sections. The three lower panels show data binned in 50 kb sections from each of the three chromosomes showing both large haploblocks in Wilder et al. (2020) and evidence for the presence of inversions from Hi-C data. Dark shades on the diagonal are indicative of high structural similarity between the reference and the Hi-C library analyzed. Dashed lines represent putative inversion breakpoints. The “butterfly pattern” of contacts observed at the point when the dashed lines meet is diagnostic of inversions.

Independent Hi-C data from a second individual from Connecticut (which was not used for genome scaffolding or heterozygosity analysis) support a high degree of accuracy in the overall assembly into chromosomes, as indicated by the strong concentration of data points along the diagonal rather than elsewhere in the contact maps (fig. 3). The contact maps also readily detected large-scale inversions (> 1 Mb) between the individual from Connecticut and the reference assembly from Georgia in three of the four chromosomes with haploblocks, i.e., 8, 18, and 24 (fig. 3 and supplementary file, Supplementary Material online). The missed detection of the inversions on chromosome 11 could either be due to their relatively smaller sizes, barely exceeding the detection threshold from Hi-C data, or because both inversion orientations segregate in Connecticut, potentially resulting in only one of the two individuals—the individual from which we generated shotgun data—carrying the ‘northern’ orientation (Wilder et al. 2020). The Hi-C-derived breakpoints of the 12.6 and 9.4 Mb inversions on chromosomes 8 and 24, respectively, matched very closely those identified by *Delly2*, although the second 2.3 Mb inversion on chromosome 24 was not supported by Hi-C data (figs. 2*a* and 3 and supplementary file, Supplementary Material online). On chromosome 18, Hi-C data showed a complex series of nested and/or adjacent inversions spanning ∼8.8 Mb in total, in contrast with the single inversion, and ∼1.3 Mb shorter, identified by *Delly2* (figs. 2*a* and 3, and supplementary file, Supplementary Material online). Additional large inversions were detected from the Hi-C data on chromosomes 4, 7, and 19. Of these, the inversion on chromosome 19 was not identified from the analysis of shotgun data from a different Connecticut individual with *Delly2*, while those on chromosomes 4 and 7 were, although with only one matching breakpoint for the inversion on chromosome 4 (figs. 2*a* and 3, and supplementary file, Supplementary Material online). Note that the identification of SVs from shotgun and Hi-C data were carried out by two different authors, and blindly from each other.

**Fig. 4.**
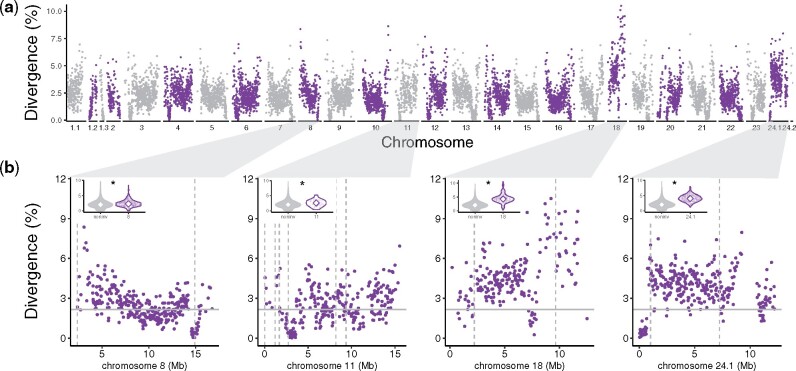
The genomic landscape of sequence divergence between Connecticut and Georgia. (*a*) Manhattan plot showing the genomic landscape of variation in divergence, where the position of each point represents the start position of an aligned sequence segment of the Connecticut genome to the Georgia reference genome on the x axis and the estimated sequence divergence across that sequence segment on the y axis. The alternating colors are used to distinguish adjacent chromosomes. The three and two scaffolds making up chromosomes 1 and 24, respectively, are represented separately here and denoted by decimal suffixes. (*b*) Enlarged Manhattan plots for each of the four chromosomes with large haploblocks and inversions. Dashed vertical line represents the breakpoints of the large inversions as identified by *Delly2* with the shotgun data and the solid horizontal line represents the sequence divergence weighted average across the genome. The small violin plots summarize and compare the distribution of sequence divergence estimates in the genomic areas not affected by large inversions (“noninv”) and areas affected by inversions in each of the four chromosomes with large haploblocks (in all comparisons sequence divergence was significantly higher in the inversion(s) in a given chromosome than in the “noninv” areas; *P *<* *0.005).

The genome-wide average sequence divergence between the Atlantic silverside genome assemblies from Connecticut and Georgia was 2.2%, but the distribution of sequence divergence was very heterogenous, resembling the distribution of heterozygosity in each genome, with similar U-shaped patterns in most chromosomes (fig. 4*a*). However, contrary to heterozygosity, which showed swaths of low heterozygosity in two of the four haploblocks, divergence was significantly higher within the inversion regions on the four chromosomes with the large haploblocks (3.3%) relative to the rest of the genome (2.1%) with much higher divergence on chromosomes 18 (4.3%) and 24 (4.1%) than on chromosomes 8 (2.3%) and 11 (2.6%; *t*-tests on weighted means, all inversions together and each inversion compared separately versus the rest of the genome, *P *<* *0.005; fig. 4*b*).

## Discussion

We generated a highly contiguous and comprehensive chromosome-level assembly of the Atlantic silverside genome. Based on karyotype information (Uwa and Ojima 1981; Warkentine et al. 1987), chromosome-level synteny with medaka, and Hi-C maps, we assigned the 27 largest scaffolds, which were longer than 1 Mb, to 24 putative chromosomes. This chromosome assembly is 88 Mb shorter than the genome size estimated through k-mer analysis, but has a lower number of duplicated genes, and only slightly fewer missing genes than the full assembly despite a substantial reduction in total sequence. If the proportion of complete genes in the chromosome assembly is a good proxy for genome completeness, then the scaffolds that are not placed in chromosomes are mostly sequences that are repetitive, redundant, or that should fill gaps in the assembled chromosomes.

Heterozygosity within a sequenced individual can result in alternative alleles getting assembled into distinct scaffolds, even in genomes much less heterozygous than the Atlantic silverside (Kajitani et al. 2014; Tigano et al. 2018), causing assembly redundancy (i.e., the same sequence being assembled into two distinct scaffolds) and thus an inflated assembly size. The final assembly, which included all scaffolds > 1 kb, was, in fact, 12% longer than the genome size estimated by the k-mer analysis. However, the long-range information provided by both the linked reads used for the 10x genome assembly draft and the proximity-ligation libraries (Chicago and Hi-C) used for further scaffolding resulted in a low proportion of duplicated genes in both the final assembly and in the chromosome assembly, indicating low redundancy. Considering the abundance of SVs between the two sequenced individuals, structural variation also may have contributed to the high number of smaller scaffolds not included in the chromosome assembly, as heterozygous SVs are notoriously hard to assemble (Huddleston et al. 2017). In the future, linkage maps and long read sequence data may help integrate the unplaced scaffolds into a chromosome assembly whose size is more similar to the estimated genome size, thereby further minimizing the effect of high levels of standing genetic variation on the assembly of the Atlantic silverside genome. Nonetheless, the current assembly adds to the increasing number of high-quality fish reference genomes, with the sixth highest contig N50 (202.88 kb) and the sixth highest proportion of the genome contained in chromosomes (84%, based on the genome size estimate from the k-mer analysis) compared to the 27 chromosome-level fish genome assemblies reported in Lehmann et al. (2019).

Patterns of synteny between the Atlantic silverside and the relatively distantly related medaka (∼91 million years) are consistent with comparisons among other teleost genomes up to hundreds of millions of years diverged: rearrangements are rare among chromosomes but common within (Amores et al. 2014; Rondeau et al. 2014; Miller et al. 2019; Pettersson et al. 2019). Consistent with this, anchoring Atlantic silverside transcriptome contigs to the medaka genome enabled the identification of four large haploblocks associated with fishery-induced selection in the lab and/or putative adaptive differences in the wild (Therkildsen et al. 2019; Wilder et al. 2020). However, the high degree of intrachromosomal rearrangements between the two species, and generally among teleosts, prevented an accurate characterization of the extent of these haploblocks and the analysis of structural variation. Differentiation, both in terms of allele frequencies and sequence divergence, between the northern and southern haplotypes seemed to extend across almost the entire length of three of the four chromosomes with haploblocks when data were oriented to medaka (Therkildsen et al. 2019; Wilder et al. 2020). Here we demonstrated that all of the four chromosomes with haploblocks carry large inversions, which seem to concentrate, and possibly maintain, these highly differentiated haplotypes. Additionally, the abundant intrachromosomal rearrangements between medaka and Atlantic silverside chromosomes shown here (fig. 1 and supplementary fig. S1, Supplementary Material online) make it clear that earlier work based on medaka genome anchoring (Therkildsen et al. 2019, Wilder et al. 2020) provided inflated impressions of the size of these inversions, which, albeit large, do not span whole chromosomes (figs. 2*a* and 3).

Our analysis of two genomes sequenced at high coverage suggested that levels of standing genetic variation, both sequence and structural, are extremely high in the Atlantic silverside. Our estimates of heterozygosity in a single individual are higher than most fish species for which data are available, including those with large census population sizes, though similar to the European sardine and two species of eels (table 2). When compared with other vertebrates, genome heterozygosity in the Atlantic silverside was more than double the highest estimate reported for birds (0.7% in the thick-billed murre *Uria lomvia*; Tigano et al. 2018) and higher than the population-based 0.6–0.9% estimates in the rabbit (*Oryctolagus cuniculus*), one of the mammals with the highest genetic diversity (Carneiro et al. 2014). Among a collection of 103 genome-wide nucleotide diversity (π) estimates (Robinson et al. 2016), only three insects and one sponge had π estimates higher than the Atlantic silverside (Corbett-Detig et al. 2015; Leffler et al. 2012). This high level of standing sequence diversity found in the Atlantic silverside and other fish species is likely due to large population sizes, with estimated *N_e_* exceeding 1 million in the European eel (Pujolar et al. 2013) and 100 million individuals in the Atlantic silverside (Lou et al. 2018), which are presumably supported by the low levels of differentiation and high population connectivity across a wide distribution range that are typical of many marine species (Tigano and Friesen 2016). As standing genetic variation is the most readily available source of adaptation to a change in the environment (Barrett and Schluter 2008), high genetic diversity in the Atlantic silverside may have facilitated the evolution of adaptive phenotypic and genetic divergence across a strong environmental cline (Hice et al. 2012; Wilder et al. 2020) and rapid responses to selection documented for the species (Conover and Munch 2002; Therkildsen et al. 2019).

Variation in nucleotide diversity across the genome has been associated with variation in recombination rates, with higher diversity and recombination rates in smaller chromosomes and in proximity of telomeres in fish, mammals and birds (Ellegren 2010; Murray et al. 2017; Sardell et al. 2018; Tigano et al. 2020, 2021). The decrease in heterozygosity from the ends towards the center of each chromosome observed in the Atlantic silverside is consistent with decreasing recombination rates as distance from the telomeres increases and has been observed in other species (Roesti et al. 2012, 2013; Haenel et al. 2018; Sardell et al. 2018). However, in addition to this U-shape pattern, heterozygosity shows a dramatic, narrow dip in each chromosome far from the center of chromosomes, suggesting a strong centromere effect. Although striking differences exist between sexes and across taxa, recombination is generally reduced or suppressed around centromeres (Sardell and Kirkpatrick 2020). The Atlantic silverside karyotype, with only four metacentric and 20 non-metacentric chromosomes (i.e., submetacentric, subacrocentric, and acrocentric; Warkentine et al. 1987), further supports that these dips in heterozygosity are associated with centromeres, as the off-center location of centromeres in non-metacentric chromosomes enable the distinction between the effect of centromeres from the effect of distance from telomeres. Although most of the narrow dips in heterozygosity are closer to the ends than to the middle of chromosomes, thus supporting the high proportion of non-metacentric chromosomes, in chromosomes 1, 18, and 24 large swaths of low heterozygosity prevent the localization of the putative centromere diagnostic dips. These patterns, combined with a coarse resolution of the karyotype features (Warkentine et al. 1987), prevent us from precisely classifying chromosomes based of position of the centromere. In forthcoming work, linkage mapping will allow us to quantify the relative effects of centromeres and telomeres on local recombination rates and ascertain whether the recombination landscape is different between sexes.

We report a 50% reduction in heterozygosity in coding sequences compared with whole-genome estimates, confirming the expectation that estimates based on exome data are not representative of whole-genome levels of standing variation. Even though the magnitude of the reduction in nucleotide diversity within coding regions is similar to levels reported in the Atlantic killifish (Reid et al. 2017) and in the butterfly *Heliconius melpomene* (Martin et al. 2016), a substantially greater reduction is seen in the collared flycatcher (86%; Dutoit et al. 2017), suggesting that the distribution of diversity in a genome, including the difference between coding and noncoding sequence, is likely idiosyncratic to the population or species examined depending on demographic factors and the strength of selective sweeps and background selection acting on coding genes. Once again, a paucity of data from other species prevents us from making generalizations, or identifying differences, on the expected reduction in diversity in coding compared with noncoding regions across taxa, while at the same time it highlights the importance of estimating and reporting basic diversity statistics for whole-genome assemblies.

We identified 4,900 SVs that survived the stringent filters applied to maximize confidence in the identified SVs and to minimize the number of false positives due to genotyping one individual only. Our estimates are likely conservative when we consider that we filtered out all heterozygous SVs, that many SVs, particularly complex ones, are hard to identify or characterize (Chaisson et al. 2019), and that we analyzed only two genomes. Nonetheless, our analyses based on shotgun data show that SVs are abundant, affect a large proportion of the genome, with inversions covering up to 23% of the genome sequence, and range in size from small (<50 bp) to longer than 10 Mb, with many of the largest inversions further supported by independent Hi-C data from a second individual. Sunflower species of the genus *Helianthus* show a similar proportion of sequence covered by inversions (22%; Barb et al. 2014), although these were detected in comparisons between species (1.5 million years diverged) rather than within species. The few studies available on other species show that structural variation tends to affect a larger portion of the genome than SNPs, but in proportions far lower than what we report here for the Atlantic silverside. For example, structural variation, including indels, duplication and inversions, affected 2.6% of the genome, three times more bases than SNPs, across six individuals of Australian snapper (*Chrysophrys auratus*; Catanach et al. 2019); in the cactus mouse (*Peromyscus eremicus*) short indels alone affected 4% of the genome of two individuals from the same population while SNPs covered only 2.3% of the genome across 25 individuals (Tigano et al. 2020); inversions, duplications and deletions combined affected 3.6% of the genome across 20 individuals of *Timema* stick insects (Lucek et al. 2019); and in Atlantic cod (*Gadus morhua*) inversions covered ∼7.7% of the genome (Wellenreuther and Bernatchez 2018 and references therein). Although levels of structural variation in the Atlantic silverside are extreme in comparison to these studies, a direct comparison with these and other species is hampered by a paucity of data and lack of common best practices for SVs genotyping (Mérot et al. 2020): comparisons similar to those made for standing sequence variation here and in other studies (e.g., Corbett-Detig et al. 2015; Robinson et al. 2016) are difficult for structural variation due to differences in sampling, approaches, data types and filtering (Mérot et al. 2020). Given the fast rate at which high-quality reference genomes are now generated, this will hopefully start to change.

The simple and affordable strategy we adopted here only requires sequencing of a single additional shotgun library prepared from a second individual—possibly from a differentiated population to capture a broader representation of intraspecific variation—and could be easily applied in other studies to start describing variation in the prevalence and genome coverage of SVs across taxa. Then, an additional Hi-C library from another individual revealed that the putative inversion on chromosome 18 was larger than indicated by the analysis of shotgun data and was actually constituted by a combination of two or more nested inversions. The apparent discrepancy between the breakpoint locations for the largest inversions identified using the two data types from two different individuals from Connecticut could reflect biological variation between the individuals analyzed. Alternatively, they may be caused by the different strengths and limitations of the underlying analytical approaches, including the fact that the identification of SVs was computational from shotgun data, while it was manually curated from Hi-C data. Although the analysis of only two individuals does not capture the full spectrum of intra- and interpopulation variation, integrating different approaches has allowed us to identify a set of high-confidence SVs to be validated and genotyped in a larger number of individuals with lower coverage data (Mérot et al. 2020).

The joint analysis of sequence and structural variation reveals interesting features of the previously identified haploblocks. The chromosome-level assembly of the Atlantic silverside genome 1) confirms that previously identified large haploblocks (Wilder et al. 2020) are associated with inversions and allows us to measure their real extent; and 2) highlights how multifaceted genomic heterogeneity can be by revealing that even haploblocks showing similar patterns of differentiation can show vastly different patterns of genetic diversity and sequence divergence. On chromosomes 18 and 24, the inversions coincide with large swaths of reduced heterozygosity (fig. 2*c*) and high sequence divergence (fig. 4*a* and *b*), which indicates that those regions were likely affected by selective sweeps or background selection thereby reducing diversity, and that these inversions maintain low diversity and high differentiation between the alleles from Connecticut and Georgia through suppressed recombination. Of note, however, the segment of chromosome 24 preceding the inversion (0–722 kb) shows an even stronger reduction in heterozygosity than the adjacent inversion (fig. 2*c*) and reduced divergence (fig. 4*b*). Although this additional reduction may be due to stronger recombination suppression in this area, perhaps associated with the presence of a centromere, the mechanism explaining this pattern remains unclear and should be further investigated. In contrast, no reduction in diversity and only modest increases, though significant, in sequence divergence are associated with the inversion on chromosome 8—the largest of them all (12.6 Mb)—and with the smaller inversions on chromosome 11 (figs. 2*c* and 4*b*). These differences between haploblocks point to idiosyncratic evolutionary histories and adaptive significance of the underlying inversions, such as differences in the ages of the inversions, the strength of selection acting on the variation captured by the inversions, the levels of gene flow between populations through time, and the demographic histories of different populations, whose investigation is now enabled by the chromosome-level genome assembly that we present here and a forthcoming analysis of population-scale whole-genome re-sequencing data. Hence, our analyses provide an empirical example of the importance of analyzing both sequence and structural variation to understand the mechanism underpinning the heterogeneous landscape of genomic diversity and differentiation.

Building on prior analysis based on in silico exome capture (Therkildsen and Palumbi 2017; Therkildsen et al. 2019; Therkildsen and Baumann 2020), this newly assembled reference genome provides an important resource for using the Atlantic silverside as a powerful model for investigating many outstanding questions in adaptation genomics, for example, related to the abundance, distribution and adaptive value of SVs; the relative role of coding and noncoding regions; the importance of sequence variation versus structural variation in both human-induced evolution and local adaptation; and the demographic and evolutionary factors generating the genomic landscape of diversity and differentiation in this and other species.

## Materials and Methods

### Reference Genome Assembly

We built a reference genome for the Atlantic silverside through three steps: First, we created a draft assembly using 10x Genomics linked-reads technology (10x Genomics, Pleasanton, CA); second, we used proximity-ligation data—Chicago^®^ (Putnam et al. 2016) and Dovetail™ Hi-C (Lieberman-Aiden et al. 2009)—from Dovetail Genomics (Santa Cruz, CA) to increase contiguity, break up mis-joins, and orient and join scaffolds into chromosomes; and finally, we used short-insert reads to close gaps in the scaffolded and error-corrected assembly. The data were generated from muscle tissue dissected from two lab-reared F1 offspring of Atlantic silversides collected from the wild on Jekyll Island, GA, USA (N 31.02°, W 81.43°; the southern end of the species distribution range) in May 2017. For 10x Genomics library preparation, we extracted DNA from fresh tissue from one individual using the MagAttract HMW DNA Kit (Qiagen). Prior to library preparation, we selected fragments longer than 30 kb using a BluePippin device (Sage Science). A 10x Genomics library was prepared following standard procedure and sequenced using two lanes of paired-end 150 bp reads on a HiSeq2500 (rapid run mode) at the Biotechnology Resource Center Genomics Facility at Cornell University. To assemble the linked reads, we ran the program *Supernova 2.1.1* (Weisenfeld et al. 2017) from 10x Genomics with varying numbers of reads and compared assembly statistics to identify the number of reads that resulted in the most contiguous assembly. Tissue from the second individual was flash frozen in liquid nitrogen and shipped to Dovetail Genomics, where Chicago and Hi-C libraries were prepared for further scaffolding. These long-range libraries were sequenced on one lane of Illumina HiSeqX using paired-end 150 bp reads. Two rounds of scaffolding with *HiRise™*, a software pipeline developed specifically for genome scaffolding with Chicago and Hi-C data, were run to scaffold and error-correct the best 10x Genomics draft assembly using Dovetail long-range data. Finally, the barcode-trimmed 10x Genomics reads were used to close gaps between contigs as the final step of the *HiRise* pipeline.

For each of the intermediate and the final assemblies we produced genome contiguity statistics using the *assemblathon_stats.pl* script from the Korf Laboratory (https://github.com/KorfLab/Assemblathon/blob/master/assemblath on_stats.pl) and assessed assembly completeness with *BUSCO v3* (Simão et al. 2015) using the Actinopterygii gene set (4,584 genes).

We estimated the genome size and heterozygosity (i.e., the nucleotide diversity π within a single individual) from the raw 10x Genomics data using a k-mer distribution approach. We removed barcodes with the program *longranger basic*, trimmed all reads to the same length of 128 bp (as read length is in the equation to estimate genome size) with *cutadapt* (Martin 2011), and estimated the distribution of 25-mers using *Jellyfish* (Marçais and Kingsford 2011). Finally, we analyzed the 25-mers distribution with the web application of *GenomeScope* (Vurture et al. 2017), which runs mixture models based on the binomial distributions of k-mer profiles to estimate genome size, heterozygosity and repeat content.

### Repeat and Gene Annotation

We annotated the Atlantic silverside genome (“final assembly”) using a combination of the *BRAKER2* (Hoff et al. 2019) and *MAKER* (Holt and Yandell 2011) pipelines, which combine repeat masking, ab initio gene predictor models and protein and transcript evidence for de novo identification and annotation of genes. To annotate repetitive elements, we first identified repeats de novo in the Atlantic silverside genome using *Repeatmodeler* (Smit and Hubley 2008) and NCBI as a search engine, and combined the resulting species-specific library with a library of known repeats in teleosts (downloaded from the RepBase website [Bao et al. 2015] in July 2018). The merged libraries were then used to annotate repeats in the Atlantic silverside genome with *Repeatmasker* (Smit et al. 2015). We then filtered annotated repeats to only keep complex repeats for soft-masking. Next, we used *BRAKER2* to train *AUGUSTUS* (Stanke et al. 2006, 2008; Buchfink et al. 2015) on the soft-masked genome with mRNA-seq evidence from 24 Atlantic silverside individuals from different populations and developmental stages, along with protein homology evidence from six different teleost species (medaka [*O.latipes*], tilapia [*Oreochromis aureus*], platyfish [*Xiphophorus maculatus*], zebrafish [*Danio rerio*], stickleback [*Gasterosteus aculeatus*], and fugu [*Takifugu rubripes*]), which were downloaded from ensemble.org (Ensembl 98; Cunningham et al. 2019) and the UniProtKB (Swiss-Prot) protein database. Second, we ran five rounds of annotation in *MAKER* using different input data sets. The first round of *MAKER* was performed on the genome with only complex repeats masked using the non-redundant transcriptome of Atlantic silverside (Therkildsen and Palumbi 2017; Therkildsen and Baumann 2020) as mRNA-seq evidence, and the six protein sequence data sets from other species as protein homology evidence. We then trained *SNAP* (Korf 2004) on the output of the initial *MAKER* run for ab initio gene model prediction. We ran *MAKER* a second time adding the SNAP ab initio gene predictions. Using the *MAKER* output from this second round, we retrained *SNAP* and ran *MAKER* three additional times (rounds 3–5) including the updated *SNAP* gene predictions, the *AUGUSTUS* gene predictions from *BRAKER2* and the updated *MAKER* annotation.

Last, we performed a functional annotation using *Blast2GO* in *Omnibox v.1.2.4* (Götz et al. 2008) using the UniProtKB (Swiss-Prot) database and *InterProScan2* (Zdobnov and Apweiler 2001) results. Annotated Atlantic silverside nucleotide sequences for all predicted genes were blasted against the UniProtKB database using *DIAMOND* v. 0.9.34 (Buchfink et al. 2015) with an *e*-value cutoff of 10^−5^. *InterProScan2* was used to annotate proteins with *PFAM* and *Panther* annotations and identify GO terms. *Blast2GO* default mapping and annotation steps were performed using both lines of evidence to create an integrated annotation file.

### Synteny with Medaka

We assessed synteny between the two species using the newly assembled Atlantic silverside reference genome from Georgia (“chromosome assembly”). We aligned the silverside genome to the medaka genome (GenBank assembly accession GCA_002234675.1) with the *lastal* program in *LAST* (Kiełbasa et al. 2011; Frith and Kawaguchi 2015) using parameters optimized for distantly related species (*-m100 -E0.05*). Given the deep divergence between the two species, we kept low-confidence alignments (*last-split -m1*). We filtered alignments shorter than 500 bp and visualized syntenic relationships using the software *CIRCA* (omgenomics.com/circa).

### Comparison of Sequence and Structural Standing Genetic Variation between Populations

As Atlantic silversides from Georgia show strong genomic differentiation from populations further north that is primarily concentrated in large haploblocks on four chromosomes (Therkildsen et al. 2019; Wilder et al. 2020), we also generated a draft genome assembly of a representative individual from Mumford Cove, Connecticut (N 41.32°, W 72.02°) collected in June 2016 for comparison. Genomic DNA was extracted from muscle tissue using the DNeasy Blood and Tissue kit (Qiagen) and normalized to 40 ng/μl. We prepared a genomic DNA library using the TruSeq DNA PCR-free library kit (Illumina) following the manufacturer’s protocol for 550 bp insert libraries. The shotgun library was sequenced using paired-end 150 bp reads on an Illumina HiSeq4000. Mate-pair libraries with insert sizes of 3, 5.3, and 8.2 kb were prepared at the Huntsman Cancer Institute at the University of Utah using the Nextera Mate Pair Library Prep Kit (Illumina) and sequenced using paired-end 125 bp reads on an Illumina HiSeq2500. We used *Trimmomatic* 0.36 (Bolger et al. 2014) to remove adapter contamination and low-quality data from both the shotgun and the mate pair libraries and used these filtered reads to assemble a draft assembly and fill assembly gaps with *Platanus v.1.2.4* (Kajitani et al. 2014) with the commands *assemble*, *scaffold*, and *gap_close*. Finally, we filtered scaffolds shorter than 1 kb.

To compare our heterozygosity estimates between Atlantic silversides from Connecticut and Georgia and with other fish species, we used two different approaches. First, we estimated genome size and heterozygosity from the raw data from the shotgun library from Connecticut using the same k-mer approach as for the Georgia individual described earlier. Then, we estimated heterozygosity directly by calculating the proportion of heterozygous sites in each genome. We used the processed 10x data as above for the Georgia individual, and the filtered shotgun data for the Connecticut individual. We mapped data from the two libraries to the chromosome assembly (only the largest 27 scaffolds—see Results) with *bwa mem* (Li and Durbin 2009) and removed duplicates with *samblaster* (Faust and Hall 2014). We called variants with *bcftools mpileup* and *bcftools call* (Danecek et al. 2014). As areas of the genome covered by more than twice the mean sequencing depth could represent repetitive areas or assembly artifact, we calculated genome coverage for each of the two libraries with *genomeCoverageBed* from *BEDtools* (Quinlan and Hall 2010) and identified the depth mode from the calculated distribution (95x for the Georgia genome and 74x for the Connecticut genome). We then filtered variants that were flagged as low-quality that had read mapping quality below 20, sequencing depth below 20, or more than twice the mode sequencing depth for each of the two libraries using *bcftools filter* (Li et al. 2009). To accurately estimate the proportion of heterozygous sites in the genome, we subtracted the number of sites that had sequencing depth below 20 and above twice the mode sequencing depth from the total genome size (to get the sum of sites that could be identified as either homozygous or heterozygous based on our criteria). Finally, we compared the Atlantic silverside estimates with those of other fish species by searching the literature for heterozygosity estimates from Genomescope with the keywords “Genomescope heterozygosity fish,” or from variant calling methods in other fish genomes, using Google Scholar.

To visualize variation along the genome, we plotted estimates of heterozygosity in 50-kb sliding windows along the genome for each of the two individuals with *ggplot2* (Wickham 2016) in R (R Core Team 2019). To assess the reduction in diversity in protein-coding regions due to positive and purifying selection, we calculated heterozygosity in the regions annotated as coding sequences only and compared this to the genome-wide estimate.

We identified SVs segregating between the Connecticut and Georgia genomes using *Delly2 v.0.8.1* (Rausch et al. 2012). For this analysis, we used the shotgun library data (74x coverage) from Connecticut mapped to the Georgia reference genome as described earlier. We called SVs using the command *delly call* and default settings. As genotyping a single individual in *Delly* is prone to false positives we applied the following stringent filters: We retained only homozygous SVs (*vac = 2*) that passed quality filters (*PASS*) and that had at least 20 reads supporting the variant calls, whether they came from paired-end clustering or split-read analysis or a combination of the two, but not more than 100 reads since these could be due to repetitive elements in the genome. As *Delly2* outputted redundant genotypes, for example inversions that had slightly different breakpoints were reported as independent variants, we used *bedtools merge* to merge these overlapping features. To validate duplication calls we also calculated coverage for each of these variants and retained only those putative duplications that had coverage more than 1.8-fold the whole-genome sequencing depth (74x).

To confirm the large SVs observed between the two genomes examined, we generated a second Hi-C library from an Atlantic silverside individual caught in Mumford Cove, Connecticut in June 2016 (a different individual than the sample used for the draft assembly). Liver tissue was excised and digested for 2 h in collagenase digestion buffer (perfusion buffer plus 12.5 μM CaCl2 plus collagenases II and IV (5 mg/ml each)). The cell suspension was then strained through a 100 μm cell strainer, washed with 1 ml cold PBS three times, resuspended in 45 ml PBS, and quantified in a hemocytometer. The cross-linking protocol was modified from Belton et al. (2012) as follows. 1.25 ml of 37% formaldehyde was added twice to the cell preparation, then incubated at room temperature for 10 min, inverting every 1–2 min. To quench the formaldehyde in the reaction, 2.5 ml of 2.5 M glycine was added three times. The sample was incubated at room temperature for 5 min, then on ice for 15 min to stop the cross-linking. The cells were pelleted by centrifugation (800 g for 10 min), and the supernatant was removed. The sample thus obtained was flash frozen in liquid nitrogen and stored at −80°C. Hi-C library preparation was performed as described previously (Belaghzal et al. 2017), except that ligated DNA size selection was omitted. 50 million fish liver cells were digested with *DpnII* at 37°C overnight. DNA ends were filled with biotin-14-dATP at 23°C for 4 h. DNA was then ligated with T4 DNA ligase at 16°C overnight. Proteins were removed by treating ligated DNA with proteinase-K at 65°C overnight. Purified, proximally ligated molecules were sonicated to obtain an average fragment size of 200 bp. After DNA end repair, dA-tailing and biotin pull down; DNA molecules were ligated to Illumina TruSeq sequencing adapters at room temperature for 2 h. Finally, the library was PCR-amplified and finalized following the Illumina TruSeq Nano DNA Sample Prep kit manual. Paired-end 50 bp sequencing was performed on a HiSeq4000. Note that the relatively low number of reads surviving filtering prevented us from further scaffolding the draft genome from Connecticut (see relatively modest number read counts and comparison with Hi-C data from Georgia on supplementary table S4, Supplementary Material online).

The two Hi-C libraries from Connecticut and Georgia (the latter prepared by Dovetail Genomics) were mapped to the Atlantic silverside chromosome assembly using the *Distiller* pipeline (github.com/mirnylab/distiller-nf). Interaction matrices were binned at 50 and 100 kb resolution and intrinsic biases were removed using the Iterative Correction and Eigenvector decomposition method (Imakaev et al. 2012). Large inversions (> 1 Mb) were identified by visual inspection of Hi-C maps as discontinuities that would be resolved when the corresponding section of the chromosomes were to be inverted (Dixon et al. 2018; Corbett-Detig et al. 2019). These discontinuities generate a distinct “butterfly pattern” with signals of more frequent Hi-C interactions where the projected coordinates of the breakpoints meet.

Finally, to estimate sequence divergence in the areas affected by large inversions on the four chromosomes with large haploblocks, we aligned the Connecticut draft genome to the Georgia reference genome using the command *nucmer* from the *Mummer4* package (Marçais et al. 2018). We filtered out alignments shorter than 10 kb with *delta-filter* and saved the divergence estimates in tabular format with the *-B* setting in *show-coords*. To compare sequence divergence within and outside the large inversions associated with haploblocks, we used the inversions breakpoint coordinates as estimated by *Delly2* from the shotgun data from Connecticut mapped to the reference genome from Georgia. We visualized variation in divergence along the genome and in each of the four chromosomes with haploblocks with *ggplot2* in R.

## Supplementary Material

Supplementary data are available at *Genome Biology and Evolution* online.

## Supplementary Material

evab098_Supplementary_Data
