# Erratum to: Chromosome-Level Assembly of the Atlantic Silverside Genome Reveals Extreme Levels of Sequence Diversity and Structural Genetic Variation

**DOI:** 10.1093/gbe/evab163

**Published:** 2021-07-31

**Authors:** Anna Tigano, Arne Jacobs, Aryn P Wilder, Ankita Nand, Ye Zhan, Job Dekker, Nina Overgaard Therkildsen

*Genome Biology and Evolution*, Volume 13, Issue 6, June 2021, evab098, https://doi.org/10.1093/gbe/evab098

Update September 9, 2021:

A previous version of this erratum incorrectly stated the author erroneously included in the originally published manuscript. This erratum has been corrected to read as follows:

When this paper was first published, Kirk Lohmueller was erroneously included in the author list. This author has now been removed from the online version. The error was introduced during the typesetting of the final article and was through no fault of the authors.

