# Supplementary material for: Chromosome-Level Assembly of the Atlantic Silverside Genome Reveals Extreme Levels of Sequence Diversity and Structural Genetic Variation
Source: Genome Biol Evol. 2021 May 8;13(6):evab098. doi: 10.1093/gbe/evab098 (PMC8214408; doi:10.1093/gbe/evab098)
Supplement: evab098_Supplementary_Data [file evab098_supplementary_data.zip › ms_meme_genomenote_suppmat_revised.docx]

**Fig. S1**. Circos plots showing synteny between the Atlantic silverside and medaka for each chromosome. Chromosomes are color-coded consistently with Figure 1 in the main text. The colored portion of the plots denotes medaka sequences, while the grey portion denotes Atlantic silverside sequences (note that the consistently shorter length of the Atlantic silverside genome is consistent with a lower overall estimate of genome size (554 Mb based on k-mer analysis compared to the 700 Mb of the assembled medaka genome). Alignments shorter than 500 bp were excluded. First page includes chromosomes 1-12, second page includes chromosomes 13-24.


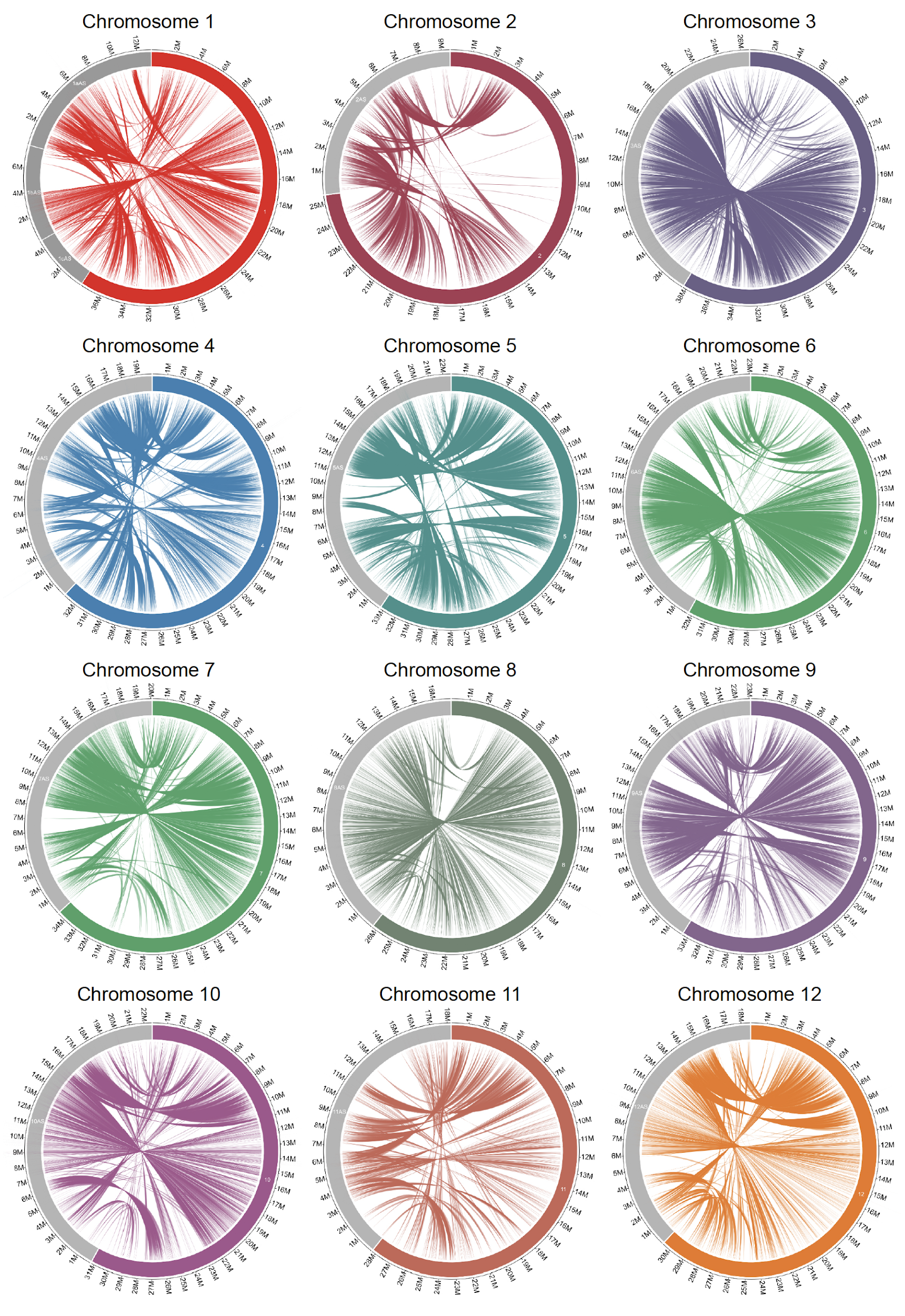


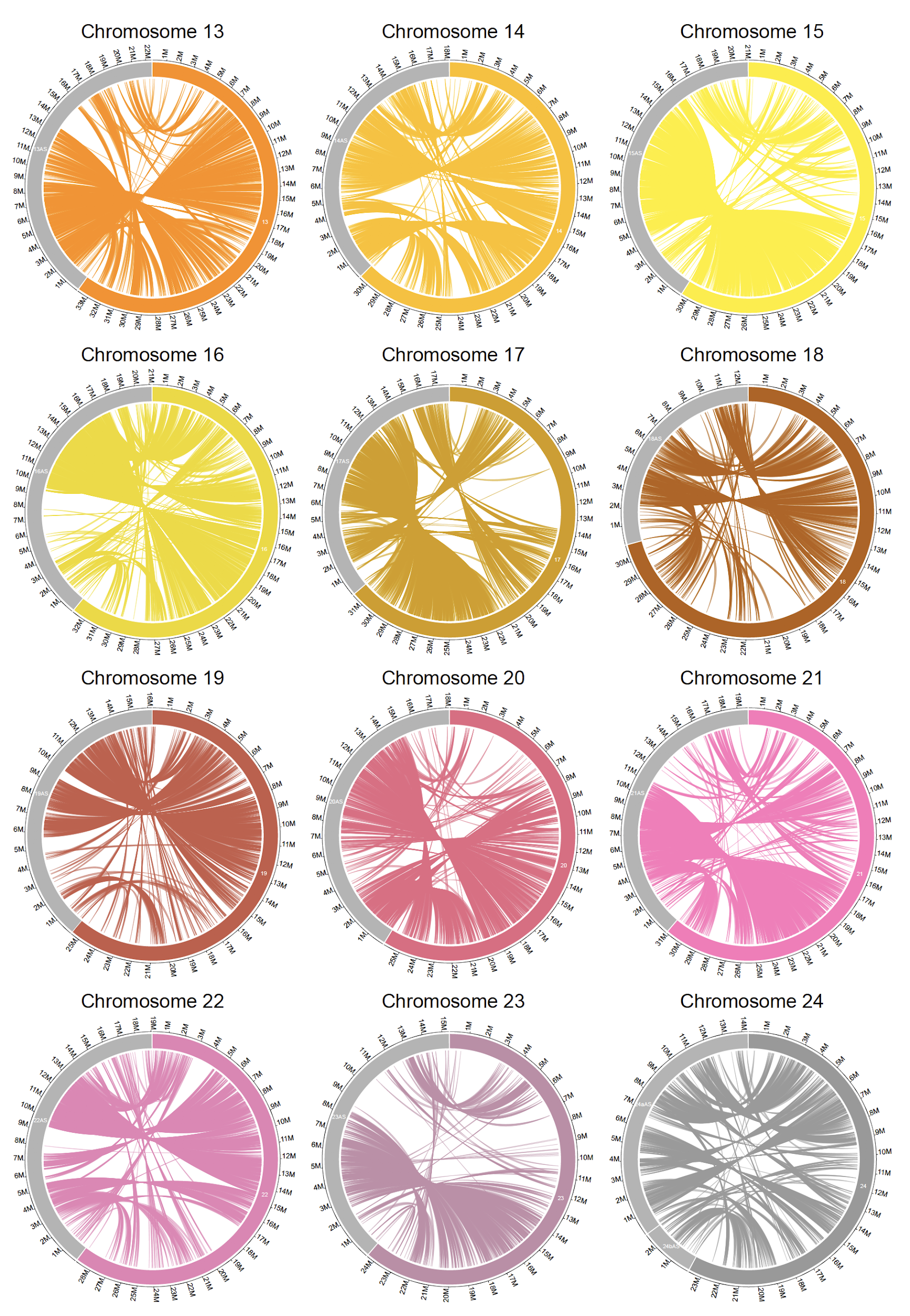


**Table S1.** Comparison of summary statistics for each draft assembly produced with 10X linked reads in Supernova using a different number of reads as input. Scaffolds are > 1 kb.

|  | **135 million** | **250 million** | **270 million** | **300 million** |
| --- | --- | --- | --- | --- |
| **# scaffolds** | 62,344 | 52,291 | 61,451 | 52,862 |
| **Size of assembly** | 504,539,299 | 598,605,697 | 618,112,803 | 618,272,469 |
| **Longest scaffold** | 8,785,170 | 12,231,131 | 12,248,921 | 12,200,847 |
| **# scaffolds > 10 Mb** | 0 | 3 | 4 | 4 |
| **N50/L50** | 617,418/141 | 1,549,464/77 | 1,527,459/73 | 1,384,316/79 |

**Table S2.** Shotgun and mate-pair reads for the assembly of the Connecticut draft genome assembly.

| **Library** | **Number of paired raw reads** | **Number of paired trimmed and quality-filtered reads** |
| --- | --- | --- |
| **Shotgun** | 352,365,972 | 233,329,623 |
| **Mate-pair 3 kb** | 81,798,806 | 49,559,804 |
| **Mate-pair 5 kb** | 125,689,645 | 58,417,565 |
| **Mate-pair 8 kb** | 104,410,162 | 60,887,372 |

**Table S3.** Summary statistics for the draft genome assembly from Connecticut (scaffolds > 1 kb).

|  | **Connecticut draft assembly** |
| --- | --- |
| **Total length** | 481.93 Mb |
| **Longest Scaffold** | 10,966,541 bp |
| **Number of scaffolds > 1kb** | 23,508 |
| **Contig N50** | 9.82 kb |
| **Scaffold L50/N50** | 71/1.671 Mb |
| **% gaps** | 10.51% |

**Table S4.** Hi-C reads from Connecticut and Georgia surviving filters for detection of large inversions.

|  | **Hi-C data from Connecticut** | **Hi-C data from Georgia** |
| --- | --- | --- |
| **Total number of reads** | 354,707,033 | 231,060,328 |
| **Unmapped reads** | 100,290,443 | 53,624,731 |
| **Single end mapped reads** | 141,973,411 | 37,688,270 |
| **Mapped reads** | 112,443,179 | 139,747,327 |
| **Duplicate reads** | 81,635,763 | 29,385,586 |
| **Unique reads** | 30,807,416 | 110,361,741 |
| **Cis reads** | 11,189,431 | 87,348,680 |
| **Trans reads** | 19,617,985 | 23,013,061 |
